# Hydrogen Sulfide—Mechanisms of Toxicity and Development of an Antidote

**DOI:** 10.1038/srep20831

**Published:** 2016-02-15

**Authors:** Jingjing Jiang, Adriano Chan, Sameh Ali, Arindam Saha, Kristofer J. Haushalter, Wai-Ling Macrina Lam, Megan Glasheen, James Parker, Matthew Brenner, Sari B. Mahon, Hemal H. Patel, Rajesh Ambasudhan, Stuart A. Lipton, Renate B. Pilz, Gerry R. Boss

**Affiliations:** 1 Department of Medicine, University of California San Diego, La Jolla, California, USA; 2 Department of Anesthesiology, University of California San Diego, La Jolla, California, USA; 3 VA San Diego Healthcare System, San Diego, California, USA; 4 Center for Aging and Associated Diseases, Helmy Institute of Medical Sciences, Zewail City of Science and Technology, Giza, Egypt; 5 Department of Chemistry and Biochemistry, University of California San Diego, La Jolla, California, USA; 6 Neurodegenerative Disease Center, The Scintillon Institute, San Diego, California, USA; 7 Beckman Laser Institute, University of California Irvine, Irvine, California, USA; 8 Department of Neurosciences, University of California San Diego, La Jolla, California, USA

## Abstract

Hydrogen sulfide is a highly toxic gas—second only to carbon monoxide as a cause of inhalational deaths. Its mechanism of toxicity is only partially known, and no specific therapy exists for sulfide poisoning. We show in several cell types, including human inducible pluripotent stem cell (hiPSC)-derived neurons, that sulfide inhibited complex IV of the mitochondrial respiratory chain and induced apoptosis. Sulfide increased hydroxyl radical production in isolated mouse heart mitochondria and F_2_-isoprostanes in brains and hearts of mice. The vitamin B_12_ analog cobinamide reversed the cellular toxicity of sulfide, and rescued *Drosophila melanogaster* and mice from lethal exposures of hydrogen sulfide gas. Cobinamide worked through two distinct mechanisms: direct reversal of complex IV inhibition and neutralization of sulfide-generated reactive oxygen species. We conclude that sulfide produces a high degree of oxidative stress in cells and tissues, and that cobinamide has promise as a first specific treatment for sulfide poisoning.

Hydrogen sulfide (H_2_S) is readily water soluble, and, at physiological pH, about two-thirds exists as hydrogen sulfide ion (HS^—^) and one-third as undissociated H_2_S^1^. We use the generic term “sulfide” to refer to both species. Sulfide is an endogenous signal transmitter via protein sulfhydration, and, at low intracellular concentrations—0.01 to 1 μM—donates electrons to complex II of the mitochondrial electron transport chain, thereby stimulating ATP production^2^,^3^,^4^,^5^,^6^. At about 3–30-fold higher concentrations, sulfide becomes toxic by binding to and inhibiting cytochrome C oxidase in complex IV of the electron transport chain, the last complex in the chain prior to ATP synthesis by complex V^3^,^4^,^5^. Cyanide, which also inhibits cytochrome C oxidase, increases mitochondrial generation of superoxide and induces oxidative stress in cells^7^,^8^. Sulfide could also be expected to induce oxidative stress, but the sulfur in sulfide is in the −2 oxidation state, the most reduced form of sulfur. Sulfide, therefore, has reducing potential—the two-electron redox potential of H_2_S is +0.17 V at pH 7—and sulfide’s effect on the cellular redox state is controversial^7^,^9^,^10^,^11^,^12^,^13^,^14^.

Workers are exposed to sulfide in many industries including agriculture, petroleum, and sewage processing, with a third of petroleum workers experiencing some symptoms from sulfide exposure and 8% having become unconscious^1^,^15^,^16^. The number of industrial deaths per year from sulfide is unknown, but sulfide is clearly a major occupational hazard, and even a one-time exposure can lead to long-term neurological deficits^17^,^18^,^19^. Sulfide can be generated easily from simple chemicals, which may explain the recent rash of sulfide-induced suicides^20^,^21^. The U.S. government considers sulfide a high priority chemical threat, both industrially and as a potential weapon of mass destruction by terrorists; its characteristic odor of rotten eggs is lost quickly due to paralysis of olfactory receptors, deceiving people of its presence^1^,^2^.

No antidote is currently available for sulfide poisoning and treatment is largely supportive. Two cyanide antidotes—hydroxocobalamin and sodium nitrite—have been tested as sulfide antidotes; hydroxocobalamin binds sulfide and nitrite generates both methemoglobin, which binds sulfide, and nitric oxide, which could displace sulfide from cytochrome C oxidase^15^,^22^,^23^,^24^. Both agents have shown some benefit in animal models, but both have to be administered either before or immediately after sulfide exposure^15^,^25^,^26^. A sulfide-poisoned person treated with hydroxocobalamin died, but he was moribund when the hydroxocobalamin was given^27^. Sodium nitrite may be beneficial in sulfide-poisoned humans, but only when administered within minutes of exposure^28^.

Cobinamide is the penultimate precursor in hydroxocobalamin (vitamin B_12_) biosynthesis by microorganisms (Supplementary Fig. 1A). We have shown it is a far better cyanide antidote than hydroxocobalamin, and recently we found it reacts readily with sulfide, neutralizing two moles of sulfide^29^,^30^,^31^. Here we compared mechanisms of sulfide and cyanide toxicity, and found that sulfide generates considerably more oxidative stress than cyanide, and that cobinamide efficiently reverses sulfide toxicity *in vitro* and *in vivo*.

## Results and Discussion

### Sulfide Inhibits Mitochondrial Respiration; Reversal by Cobinamide

Sodium hydrogen sulfide (NaSH) is used commonly as a sulfide source, because it is stable, and much easier to work with than hydrogen sulfide gas^3^,^4^,^5^,^11^,^14^. Using NaSH, we studied sulfide toxicity in four cell types: hiPSC-derived cortical neurons, primary rat cortical neurons, primary human fibroblasts, and COS-7 monkey kidney cells. We studied neurons, because the brain is a major sulfide target—as evidenced by both acute toxicity (coma) and long-term sequelae of exposure (cognitive and motor deficits), comparing results to the other, faster proliferating cell types.

**Figure 1:**
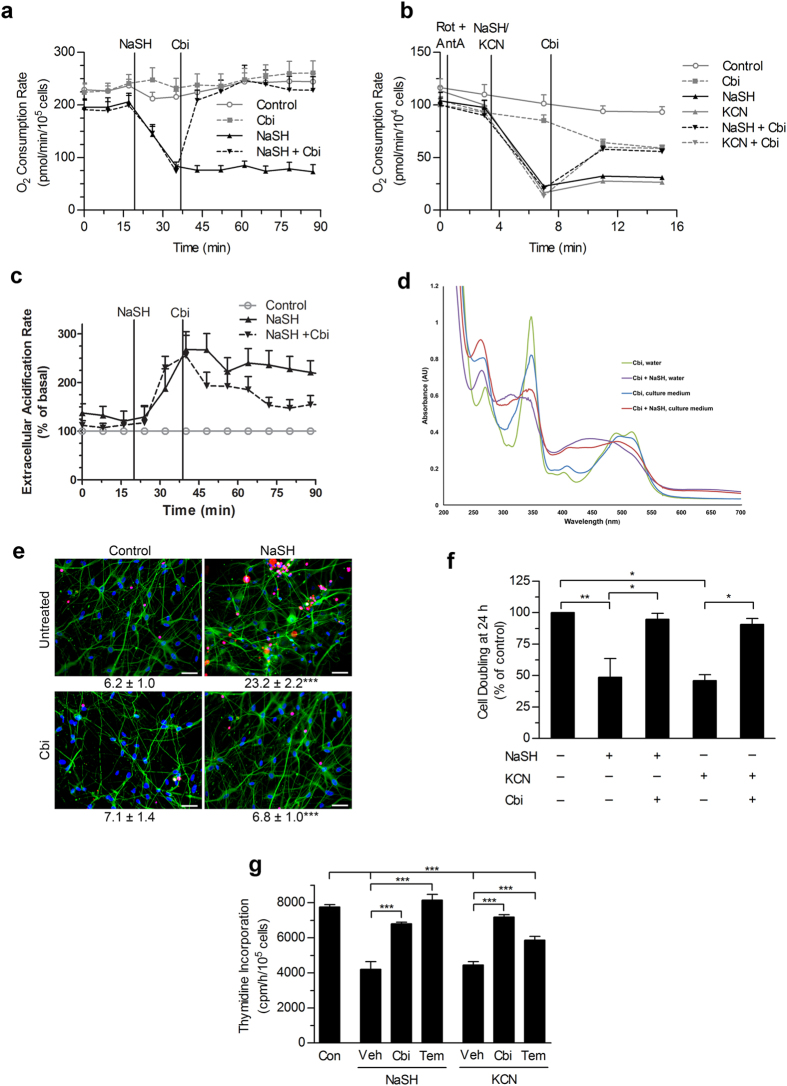
Cobinamide Rescue of Cellular Sulfide Toxicity. (**a**) Oxygen consumption was measured in hiPSC-derived neurons; 1 mM NaSH and/or 100 μM cobinamide (Cbi) were added as indicated. Control cells (circles, solid grey line); cobinamide-treated cells (squares, dashed grey line); NaSH-treated cells (triangles, solid black line); NaSH-treated cells subsequently treated with cobinamide (triangles, dashed black line). (**b**) Permeabilized human fibroblasts were incubated with TMPD, a complex IV substrate; 2 μM rotenone, 4 μM antimycin A, 0.5 mM NaSH or KCN, and/or 100 μM cobinamide were added as indicated. Oxygen consumption was measured as in **a**, but reflects largely complex IV activity. Symbols are as in (**a**) except KCN-treated cells (triangles, solid grey line) and KCN-treated cells that received cobinamide (triangles, dashed grey line) are shown. **(c)** Extracellular acidification was measured in human fibroblasts, with 1 mM NaSH and 100 μM cobinamide added as indicated. Symbols are as in (**a**). (**d**) Cobinamide (1 mM) was added to water or DMEM culture medium with or without an equimolar amount of NaSH. The samples were diluted 20-fold and their UV-visible spectra recorded. (**e–g**) hiPSC-derived neurons (**e**) or human fibroblasts (**f,g**) received 1 mM NaSH or 1 mM KCN for 2 h, with some cells receiving 400 μM cobinamide or 200 μM 4-hydroxy-TEMPO (Tem) during the second hour. The cells were washed and incubated in non-drug-containing medium. (**e**) At 20 h, cells were stained with DAPI (nuclei, blue) and β3-tubulin (green); TUNEL-positive, apoptotic cells were stained red. Numbers below the photographs are the percent of apoptotic cells (mean ± SEM); scale bars are 50 μm. (**f**) At 24 h, cells were counted, and cell doublings are shown as a percent of control cells. (**g**) At 24 h, DNA synthesis was measured. Con, control, Veh, vehicle. The data are the means ± SEM of three independent experiments performed in triplicate. In (**a**–**c**) the difference between cobinamide-treated and NaSH or KCN only was significant within 8 min post cobinamide addition (*P* < 0.05). In **e**, ***P < 0.001 for comparison to control or NaSH-treated cells, and in **f** and **g,** *P < 0.05, **P < 0.01, ***P < 0.001 for indicated comparisons.

Cellular oxygen consumption provides a good measure of mitochondrial function, and we found that 1 mM NaSH rapidly reduced oxygen consumption in hiPSC-derived cortical neurons, primary human fibroblasts, and COS-7 monkey kidney cells (Fig. 1a, Supplementary Fig. 1b,c). The reduction in oxygen consumption was similar to that by an equimolar concentration of cyanide, and studies with selective complex I and III inhibitors pointed to inhibition of complex IV of the mitochondrial respiratory chain (Fig. 1b, Supplementary Fig. 1d). These results are consistent with those of previous workers^3^,^4^,^5^. Concomitant with the fall in oxygen consumption, sulfide increased cellular acid production (Fig. 1c, Supplementary Fig. 1e), likely from increased lactate generation due to a switch to anaerobic metabolism; these studies could not be done in the neuronal cells, because they could not tolerate medium with low buffering capacity, which is required for measuring extracellular acidification. We found that 100 μM cobinamide, which alone had no effect on oxygen consumption rates, quickly reversed the inhibition of oxygen consumption and complex IV activity induced by sulfide and cyanide, and reversed the sulfide-induced increase in acid production (Fig. 1a–c, Supplementary Fig. 1b–e). Cobinamide’s rapid and full reversal of sulfide-induced changes suggest cobinamide removes sulfide from complex IV. We have shown that cobinamide reacts rapidly with sulfide in biological buffers—yielding SSH_2_^—^ in a stable complex with cobinamide—and, thus, each cobinamide molecule neutralizes two sulfide molecules^31^. We now found that cobinamide reacts readily with sulfide in tissue culture medium, which simulates a more physiological condition (Fig. 1d; the spectral difference between water and the culture medium is due to cobinamide binding to amino acids and other medium constituents).

Up to 100 μM, cobinamide is well tolerated by several different types of cells, having a minimal effect on cell growth over a 72 h period^32^. For comparison, we found that hydroxocobalamin, which binds sulfide and has been suggested as a sulfide antidote, also reversed sulfide inhibition of oxygen consumption, but it was considerably less potent than cobinamide: in human fibroblasts, hydroxocobalamin did not fully reverse the effect of sulfide, and, in COS-7 cells, 10-fold more hydroxocobalamin than cobinamide was required to reverse sulfide’s inhibitory effect (Supplementary Fig. 1b,c).

### Sulfide Is Toxic to Cells; Rescue by Cobinamide

Consistent with clinical data that short sulfide exposures can lead to subsequent long-term neurological deficits, we found that a 2 h exposure to 1 mM NaSH markedly increased apoptosis in hiPSC-derived neurons and primary rat neurons, and reduced cell proliferation and DNA synthesis in fibroblasts when assessed 20–72 h later (Fig. 1e–g, Supplementary Fig. 1f,g; we could not measure proliferation and DNA synthesis in the neuronal cells due to their slow growth rate). Sulfide inhibition of cell proliferation and DNA synthesis was similar to that which occurred with an equimolar concentration of cyanide (Fig. 1f,g). At 400 μM, cobinamide prevented the increase in apoptosis and returned cell proliferation and DNA synthesis to normal when present during the second hour of sulfide exposure, simulating a real-life scenario of treatment initiated after the onset of sulfide exposure (Fig. 1e–g, Supplementary Fig. 1f,g). Once again, hydroxocobalamin was not as effective as cobinamide at reversing sulfide-induced toxicity, since twice as much hydroxocobalamin as cobinamide was required to allow full cellular proliferation in the presence of sulfide (Supplementary Fig. 1g).

### Mechanism of Sulfide Cellular Toxicity

Sulfide’s inhibition of complex IV of the mitochondrial electron transport system could cause cellular toxicity via reduced ATP production and/or generation of oxidative stress.

#### Cellular ATP Concentration

We found no significant change in the intracellular ATP concentration after 2 h of incubation with 1 mM NaSH, the same amount of sulfide exposure that increased apoptosis and inhibited DNA synthesis (Table 1; a trend towards reduced ATP in the hiPSC cortical neurons did not reach statistical significance). Although these results may seem surprising, the increased acid production we observed during sulfide exposure likely reflected increased glycolysis, which could compensate for the loss of mitochondrial function. Moreover, the intracellular ATP concentration is relatively high, i.e., 2–4 mM, and would not necessarily change after short periods of decreased mitochondrial respiration. We also found no significant change in the intracellular ATP concentration in cyanide-treated cells (Table 1), which again was likely due to a switch to anaerobic metabolism, since lactic acidosis is a hallmark of cyanide poisoning^33^. Few studies have measured ATP in sulfide- or cyanide-exposed cells or tissues, but sulfide and cyanide reduced ATP in rat thoracic aortic rings pre-constricted with epinephrine^34^, presumably, because of the high ATP consumption during contraction.

#### Oxidative Stress

**Figure 2:**
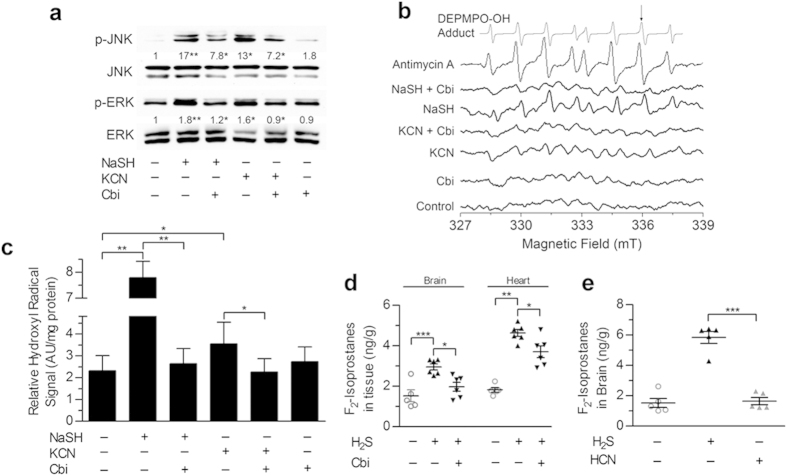
Cobinamide Reverses Sulfide-Induced Oxidative Stress. (**a**) Human fibroblasts received 1 mM NaSH or KCN for 2 h; some cells received 200 μM cobinamide during the second hour. Phospho-JNK (p-JNK) and phospho-ERK (p-ERK) were analyzed in cell extracts by immunoblotting and quantified by densitometric scanning. The numbers are means of three independent experiments performed in triplicate; the NaSH- and KCN-treated samples are compared to the control, and the cobinamide-treated samples are compared to the same condition lacking cobinamide. (**b**) Mitochondria were isolated from mouse hearts, and exposed for 5 min to 200 μM NaSH or KCN, with or without 100 μM cobinamide. The EPR signal from 327 to 349 milliTesla was recorded with DEPMPO serving as a spin probe. The reciprocal shape of the spectra on either side of ~333 milliTesla is from hyperfine coupling between the free radical adduct and the heteronuclear spins of the hydrogen, nitrogen, and phosphorus atoms of DEPMPO. We included 4 μM antimycin A as a positive control, and show a computer-calculated spectrum of the DEPMPO-OH adduct. **(c)** Data from three independent experiments conducted as in (**b**) are shown for the DEPMPO-OH adduct labeled with an arrow in (**b**) (values are mean ± SEM). NaSH values are plotted on the upper part of the broken “Y” axis, with the other conditions plotted on the lower part of the “Y” axis. (**d**) Mice were exposed to a sublethal dose of H_2_S gas (1270 ppm for 15 min); some mice received an intramuscular injection of 375 μmol/kg cobinamide 5 min prior to gas exposure. The mice were euthanized; their brains and hearts were flash frozen, and extracted and analyzed for F_2_-isoprostanes by mass spectrometry. (**e**) Mice were exposed to lethal doses of H_2_S or cyanide gas (1270 and 584 ppm, respectively, for 40 min); immediately after death, brain F_2_-isoprostanes were measured. Cyanide has a lower LD_100_ than sulfide^1^,^51^ For panels (**d**,**e**) each symbol represents one mouse; the large and small horizontal lines show the mean and SEM, respectively (n = 5–6). For all panels, *P < 0.05, **P < 0.01, and ***P < 0.001 for indicated comparisons.

Sulfide has been reported to both reduce and increase oxidative stress in cells and whole animals^7^,^9^,^10^,^11^,^12^,^13^,^14^. The protein kinases JNK and Erk are activated when cells are stressed by a variety of agents, including reactive oxygen species^35^. At 1mM, NaSH activated both kinases to a greater degree than an equimolar concentration of cyanide; 200 μM cobinamide partially returned JNK and fully returned Erk activation/phosphorylation to that of control cells (Fig. 2a).

In isolated mouse heart mitochondria, 200 μM NaSH increased the generation of hydroxyl radical more than three-fold, one of the most damaging reactive oxygen species [Fig. 2b,c^36^]. The sulfide-induced increase in hydroxyl radical was more than twice that induced by an equimolar amount of cyanide (Fig. 2b,c). For both sulfide and cyanide, 100 μM cobinamide returned hydroxyl radical generation to that of control mitochondria (Fig. 2b,c). Cobinamide’s reduction of hydroxyl radical in sulfide- and cyanide-exposed cells could be from binding sulfide and cyanide; however, we found that cobinamide also neutralizes superoxide anion, presumably by a redox reaction generating oxygen and reduced cobinamid e [Supplementary Fig. 2 37]. Free radical neutralization may be a major mechanism whereby cobinamide reversed the cellular toxicity of sulfide and cyanide, because we found that 4-hydroxy-TEMPO, a scavenger of reactive oxygen species, also reversed sulfide- and cyanide-induced inhibition of DNA synthesis (Fig. 1g).

Exposing mice to a sublethal dose of H_2_S gas (1270 ppm for 15 min), increased F_2_-isoprostanes almost two-fold in brain and more than three-fold in heart, two major target organs of sulfide (Fig. 2d). F_2_-isoprostanes are derived non-enzymatically by peroxidation of arachidonic acid, and are an excellent measure of reactive oxygen species generation in tissues^38^; thus, the data indicate that sulfide generated substantial oxidative stress *in vivo*. Administering cobinamide immediately before H_2_S gas exposure at a dose that is safe in mice significantly reduced both brain and heart F_2_-isoprostanes (Fig. 2d). Increasing the H_2_S gas exposure time to 40 min—a lethal H_2_S dose—increased tissue F_2_-isoprostanes further, while exposing mice to a lethal dose of cyanide gas did not increase tissue F_2_-isoprostanes significantly (Fig. 2e).

Thus, both sulfide and cyanide induced cellular stress, with sulfide generating considerably more oxidative stress than cyanide; cobinamide reduced the cellular and oxidative stress induced by both poisons.

### Cobinamide Rescues Flies and Mice Exposed to H_2_S Gas

We have shown that cobinamide rescues rabbits from a lethal intravenous infusion of NaSH^39^. Although NaSH infusion is a common method to poison animals with sulfide, the primary mode of human sulfide exposure is via inhalation of H_2_S gas, which could lead to different pathophysiological changes than NaSH infusion. We, therefore, designed two separate animal models of H_2_S gas exposure, based on our previous experience with cyanide gas^29^,^30^.

**Figure 3:**
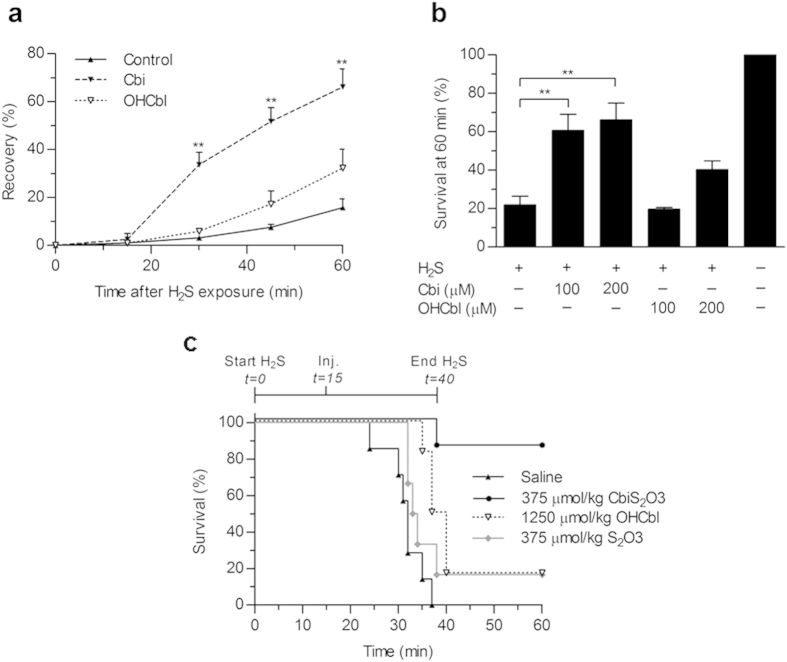
Cobinamide Rescues Flies and Mice Exposed to H_2_S Gas. (**a**) *D. melanogaster* were exposed to H_2_S gas for 20 min, falling motionless within 1–2 min of gas exposure. They were transferred to ambient air, and monitored every 15 min for 1 h for recovery, defined as the ability to walk or fly. Some of the flies had been fed food containing 200 μM cobinamide (dashed line) or 200 μM hydroxocobalamin (dotted line). (**b**) Flies were treated as in Panel (**a**) with recovery recorded at 60 min. Some flies had received food containing the indicated concentrations of cobinamide or hydroxocobalamin. Data in (**a**,**b**) are shown as mean ± SEM of three and five independent experiments, respectively, with at least 20 flies per condition in each experiment; **P < 0.01 compared to control flies. (**c**) Mice were exposed to 1270 ppm H_2_S gas for 15 min in a gas-tight chamber, and then removed from the chamber and injected with the indicated agent intraperitoneally; they were then placed back in the chamber and re-exposed to H_2_S gas for another 25 min. Mice that survived for 60 min remained alive and well until one week later when they were euthanized (*n* = 5–6 per condition). Six mice were studied for each condition, and the difference between the cobinamide-treated and saline-treated animals was statistically different (*P* < 0.05). Cbi, cobinamide; OHCbl, hydroxocobalamin; CbiS_2_O_3_, cobinamide thiosulfate; S_2_O_3_, thiosulfate.

First, we used *D. melanogaster*, which are being used increasingly in drug discovery^40^. We found that within one to two minutes of exposing flies to H_2_S, the flies fell motionless; this recapitulates the rapid “knock-down” and unconscious state experienced by humans exposed to H_2_S gas^1^,^15^. After 20 min of H_2_S exposure, the flies were transferred to ambient air, and, over the ensuing hour, ~20% of flies recovered, with few flies recovering beyond that time (Fig. 3a). In contrast, ~65% of flies that had been grown on food containing 100 or 200 μM cobinamide recovered from the H_2_S exposure, while flies grown on food containing 100 or 200 μM hydroxocobalamin showed either no improvement compared to control flies or a 40% recovery rate, respectively (Fig. 3a,b). The improved recovery of cobinamide-fed flies compared to hydroxocobalamin-fed flies was not due to greater cobinamide absorption, because the two drugs yielded similar concentrations of ~2 μM; exclusive of the heads, total body cobinamide and hydroxocobalamin concentrations were measured (Supplementary Fig. 3).

In the second animal model, we exposed mice to a lethal concentration of H_2_S gas in an enclosed chamber for 15 min, and then removed the mice and injected them intraperitoneally with the test agent; we placed them back in the chamber and re-exposed them to H_2_S gas for another 25 min. This model simulates a real-life scenario of people exposed to H_2_S gas, such as in an industrial accident or terrorist attack, with about 15 min required for emergency medical personnel to arrive at the scene, and another 25 min to treat and evacuate the victims. All mice treated with saline died between 25 and 37 min, i.e., 10–22 min into the second H_2_S exposure period (Fig. 3c). This is to be contrasted with an 83% survival rate in mice treated with cobinamide at the same non-toxic dose used in the non-lethal model described previously (Fig. 3c). Cobinamide was administered as a thiosulfate derivative, because aquohydroxocobinamide is not well absorbed and a ligand needs to be bound to the cobalt atom for efficient absorption^30^. The same amount of thiosulfate by itself yielded a 16.7% survival rate (Fig. 3c); thiosulfate was previously reported to have a modest antidotal effect against sulfide through an unknown mechanism^15^. Hydroxocobalamin at more than three times the cobinamide dose yielded only a 16.7% survival rate (Fig. 3c). The considerably greater efficacy of cobinamide over hydroxocobalamin, both in cell studies as well as in animals, could be from cobinamide having a higher affinity for sulfide and/or from cobinamide’s ability to neutralize reactive oxygen species^31^.

## Conclusions

While sulfide and cyanide both inhibit cytochrome C oxidase, and would, therefore, be expected to generate superoxide, we found that sulfide induces considerably more oxidative stress than cyanide. This could be because sulfide can react directly with oxygen to form reactive oxygen species, and/or because sulfide can serve as a substrate for complex II of the mitochondrial electron transport chain, thereby providing electrons to mitochondria^3^,^4^,^41^,^42^. Cobinamide, a drug in the advanced stages of development for cyanide poisoning, reduced sulfide toxicity and sulfide-induced oxidative stress in cells and whole animals, showing promise as a novel and first specific therapy for sulfide poisoning. Cobinamide has a half-life of about 10 h in animals, and its main mode of elimination appears to be through renal excretion; its relatively long half-life implies it could be used prophylactically, in addition to therapeutically. Cobinamide is safe in mice at doses several-fold higher than those used in these studies, with development of coagulation abnormalities as the dose-limiting effect.

## Methods

### Materials

Hydroxocobalamin was from Molekula. Aquohydroxocobinamide, referred to as cobinamide throughout the text, was produced by base hydrolysis of hydroxocobalamin using cerium hydroxide as described previously, and was >98% pure as assessed by high-performance liquid chromatography^43^. It is water soluble up to concentrations of 300 mM, with standard stock solutions made at 100 mM. Sodium hydrogen sulfide (NaSH) was from Alfa Aesar and was stored in a vacuum dessicator; under this condition, the product was stable for several months^44^. Potassium cyanide, *N,N,N’,N’*-tetramethyl-*p*-phenylenediamine (TMPD), and 4-hydroxy-2,2,6,6-tetramethylpiperdine-1-oxyl (4-hydroxy-TEMPO) were from Sigma-Aldrich; 5-(diisopropoxyphosphoryl)-5-ethyl-1-pyrroline-N-oxide (DEPMPO) was from Enzo Life Sciences. The Erk 1 antibody was from Santa Cruz Biotechnology (SC-93), and p-Erk, p-JNK, and JNK antibodies were from and Cell Signaling (4376S, 9251S, and 9252S, respectively).

### Cell Origin and Culture

We studied hiPSC-derived cortical neurons and primary rat cortical neurons, because the central nervous system is a major target of sulfide poisoning. The neuronal cells are terminally differentiated and do not proliferate, and because we were interested in studying the effect of sulfide on proliferating cells, we also studied fibroblasts and fibroblast-like cells; the latter cells also allowed us to determine if the results observed in neuronal cells occurred in other cell types.

Human induced pluripotent stem cell (hiPSC)-derived cerebrocortical neurons were generated from human dermal fibroblasts using an integration-free reprogramming method. Fibroblasts were electroporated with episomal expression vectors encoding six reprogramming factors: OCT3/4, SOX2, KLF4, L-MYC, LYN28, and p53-shRNA using a Lonza nucleofector system^45^. hiPSC colonies were maintained on mouse embryonic fibroblast feeders, and were validated for pluripotency, trilineage differentiation capability, and karyotypic stability as described^46^. Neuronal differentiation was performed using a protocol modified from Chambers *et al.*^47^. Briefly, feeder-free hiPSCs were treated with small-molecule inhibitors of bone morphogenetic protein (Dorsomorphin), Activin/Nodal (A83-01), and Wnt/β-catenin (PNU-74654) for 5 d. The cells were cultured for 2–3 wk as floating neurospheres in DMEM/F12 medium supplemented with N2 and B27 (Invitrogen) in the presence of basic FGF (20 ng/ml). They were allowed to form a monolayer on p-ornithine/laminin-coated dishes, and resulting PAX6 + neural rosettes were manually isolated and expanded. The cells were seeded onto glass coverslips (7 × 10^5^ cells/cm^2^) for terminal differentiation into mature neurons, over a period of 2 months, in the presence of medium supplemented with N2, BDNF (10 ng/ml), GDNF (10 ng/ml), and dibutyryl-cAMP (0.5 mM). Prior to experimentation, the neurons were characterized as described^48^.

Primary cerebrocortical neurons were isolated from E16-18 Sprague-Dawley rats, and maintained in culture as described^49^. Briefly, rat cerebrocortex was dissected and incubated with trypsin. Dissociated cells were cultured in D10C medium plated on poly-l-lysine-coated plates. Experiments were performed 14–21 d after initial plating.

Primary human fibroblasts and monkey kidney fibroblast-like cells (Cos-7 cells) were obtained from the American Type Culture Collection (CRL-1501 and CRL-1651, respectively), and were grown in minimal essential medium and Dulbecco’s modified Eagle’s medium (DMEM), respectively; both media were supplemented with 10% fetal bovine serum. Both cell lines were negative for mycoplasma infection by the MycoAlert^TM^ Mycoplasma Detection Kit (Lonzo Group).

### Measurement of Rates of Cellular Oxygen Consumption and Extracellular Acidification

hiPSC-derived neurons at four weeks into terminal differentiation were seeded on XF24 plates (Seahorse Bioscience, North Billerica, MA) at 750,000 cells/well, and cultured for at least two weeks; immediately prior to assay, the cells were placed in culture medium containing 40 mM HEPES and 0.4% fatty-acid free bovine serum. Human fibroblasts and COS-7 cells were seeded on XF24 plates at 40,000 cells/well and cultured overnight; one hour prior to assay, the cells were placed in unbuffered XF assay medium. Rates of cellular oxygen consumption and extracellular acidification were measured using a Seahorse XF24 Extracellular Flux analyzer; agents were administered to the cells through automated injection ports. Measurement of extracellular acidification could not be done in the in hiPSC-derived neurons, because they could not withstand the required unbuffered assay medium.

### Measurement of Mitochondrial Complex IV Respiration

Human fibroblasts and COS-7 cells were seeded on XF24 plates at 40000 cells/well and cultured overnight. The cells were incubated in a mitochondrial assay solution containing 1.0 nM XF plasma membrane permeabilizer (Seahorse Bioscience), 4 mM ADP, 10 mM pyruvate, 0.5 mM malate and 4 mM TMPD. At the indicated times in the figures, 2 μM rotenone and 4 μM antimycin A (mitochondrial complex I and III inhibitors, respectively) were injected followed by test agents.

### Recording of UV-Visible Spectra

The UV-visible spectrum of cobinamide in the absence and presence of NaSH was recorded on a Kontron 960 spectrophotometer.

### Measurement of Apoptosis, Cell Proliferation, and DNA Synthesis

Cells in logarithmic growth received 1 mM NaSH or 1 mM KCN for 2 h, with 400 μM cobinamide or 200 μM 4-hydroxy-TEMPO added to some of the cells during the second hour of NaSH exposure. The cells were washed twice with phosphate-buffered saline (PBS) and fresh growth medium was added.

Apoptosis was assessed 20 h later using an *in situ* cell staining kit based on the TUNEL assay (Roche Applied Science). TUNEL positive cells were counted in six areas of four different wells per condition, counting at least 1000 cells per condition.

Cell proliferation was determined by counting cells at 24, 48 and 72 h post NaSH exposure using a TC20 Automatic Cell Counter (Bio-Rad, Hercules, CA). The number of cell doublings (CD) was calculated according to the formula:


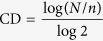


where N is final cell number, and n is initial cell number.DNA synthesis was measured 24 h post NaSH exposure by incubating cells for 1 h with 5 μCi [methyl-^3^H]thymidine (20 Ci/mmol, final concentration 0.5 μM). The cells were washed in PBS and extracted *in situ* in 10% (w/v) trichloroacetic acid. Precipitated DNA was collected on glass microfiber filters; radioactivity on the filters was measured by liquid scintillation counting. Measurement of cell proliferation and DNA synthesis was done in the fibroblasts only, because the hiPSC-derived neurons and the primary rat neurons grew too slowly for these assays.

### Measurement of Intracellular ATP Concentration

Cells in logarithmic growth were treated with 1 mM NaSH or 1 mM KCN for 30, 60, and 120 min, and were then rapidly extracted *in situ* in ice-cold 0.4 N perchloric acid. The extracts were centrifuged, the supernatants were neutralized with KHCO_3_ and precipitated potassium perchlorate was removed by centrifugation. ATP was measured in the neutralized extracts by the firefly luciferase method. Absolute amounts of ATP were determined by comparison to standard curves using freshly prepared ATP.

### Assessment of JNK and Erk Activation

Human fibroblasts received 1 mM NaSH or 1 mM KCN for 2 h, with or without 200 μM cobinamide added during the second hour. The cells were lysed *in situ*, and the lysates were subjected to polyacrylamide gel electrophoresis. Proteins were identified by Western blotting using antibodies against p-Erk, Erk-1, p-JNK, and JNK.

### Measurement of Mitochondrial Generation of Reactive Oxygen Species

Male C57BL/6J mice aged 8–12 weeks were euthanized, and their hearts were removed quickly; all subsequent steps were performed at 4°C. The ventricles were cut away from the heart, and homogenized in mitochondrial isolation medium (0.3 M sucrose, 10 mM HEPES, 250 μM EDTA), initially using a Tissuemiser (Fisher Scientific, Waltham, MA, USA) and then a potter homogenizer. Mitochondria were isolated by differential centrifugation and incubated in mitochondrial respiration buffer containing malate and pyruvate; some samples were treated with 200 μM NaSH or KCN, with or without 100 μM cobinamide for 5 min. The samples were mixed with the spin trap DEPMPO (70 mM), loaded into glass capillary tubes, and the electron paramagnetic (EPR) signal from 327–349 milliTelsa was measured in a MiniScope MS300 benchtop spectrometer (Magnettech GmbH, Berlin, Germany). Assignment of observed signals was confirmed through computer-assisted spectral simulation using WinSim software (http://epr.niehs.nih.gov/pest.html). Signals were quantified by measuring peak amplitudes of observed spectra, and normalized to mitochondrial protein.

### Assessment of Cobinamide Reaction with Superoxide Anion

Superoxide anion radical was generated *in vitro* using a hypoxanthine-xanthine oxidase system, and detected by electron paramagnetic resonance (EPR) using DEPMPO as a spin trap. Varying concentrations of cobinamide were added to the system.

### Measurement of Brain and Heart F_2_-Isoprostane Content

Male C57BL/6J mice, 8–12 weeks old, were placed in an airtight gas chamber, and anesthetized by injecting isoflurane into the chamber to a final concentration of 2%. The anesthetized mice were then exposed to 1270 ppm H_2_S gas (generated as described below) for either 15 min (a sub-lethal dose) or 40 min (a 100% lethal dose). Some of the animals received an intraperitoneal injection of cobinamide thiosulfate immediately before being placed in the chamber. To compare sulfide to cyanide exposure, some mice were exposed for 40 min to 584 ppm cyanide gas, a 100% lethal dose^50^. Mice that received the sublethal dose of H_2_S were euthanized, and the hearts and brains from all mice were removed quickly, flash frozen in liquid nitrogen, and stored at −80 °C. The samples were extracted, and F_2_-isoprostanes were measured by gas chromatography-mass spectrometry at the Vanderbilt University Eicosanoid Core Laboratory^38^.

### Exposure of *D. Melanogaster* to Hydrogen Sulfide Gas

Wild type Oregon R *D. melanogaster* were grown on control food, or food containing 100 or 200 μM cobinamide or hydroxocobalamin, throughout their life cycle as described previously^29^. The flies were transferred to 10 ml vials, and H_2_S gas was generated in the vial by spotting 10 μl of 2 M NaSH on a 0.5 × 0.5 cm square of Whatman filter paper preloaded with 10 μl of 5 M H_2_SO4. The vial was sealed tightly, and 20 min later, the paper was removed and the flies were transferred to hydrogen sulfide-free vials. The flies were monitored for activity every 15 min for 1 h, and those able to walk or fly were considered recovered.

### Measurement of Cobinamide and Hydroxocobalamin in *D. Melanogaster*

Flies were anesthetized on ice and decapitated. The bodies were homogenized in 0.4 N perchloric acid, the samples were centrifuged, and the supernatant was neutralized with KHCO_3_. KCN was added to the samples to generate dicyanocobinamide or cyanocobalamin, respectively. The amount of dicyanocobinamide and cyanocobalamin was quantified by high performance liquid chromatography as described previously^43^. The cobinamide or hydroxocobalamin concentration in the flies was calculated using an estimated fly body volume of 2 μl.

### Exposure of Mice to Hydrogen Sulfide Gas

Male C57BL/6J mice, 8–12 wks old, were anesthetized with isoflurane in an airtight gas chamber as described previously^30^. Hydrogen sulfide gas was generated in the chamber to a concentration of 1270 ppm by injecting 1 ml of 500 mM NaSH into a glass beaker containing briskly-stirred 1 M sulfuric acid; the chamber is equipped with a fan, which rapidly equilibrates the generated gas. The mice were exposed to the gas for 15 min, and then removed from the chamber and given an intraperitoneal injection of test agent. They were placed back in the chamber for 25 min more of H_2_S gas exposure, for a total gas exposure time of 40 min. They were removed from the chamber and surviving animals were observed for 1 wk. A sample size of six in each group was determined by a Chi-square test, assuming 100% lethality in control animals and at least 80% survival in treated animals, at an alpha value of 0.05. No animals were excluded from the study, and, although the animals were not formally randomized, each experimental day animals were randomly assigned to receive one of four experimental treatments. The study could not be performed in a blinded fashion, because cobinamide and hydroxocobalamin are intensely colored substances, and the operator was, therefore, aware of the test agent.

### Study Approval

The mouse studies were approved by the University of California, San Diego IACUC (hydrogen sulfide and cyanide exposure) and the VA San Diego Healthcare System IACUC (isolation of heart mitochondria). Both facilities are AAALAC accredited, and the mice were cared for and treated according to AAALAC standards.

### Statistics

Data are reported as means ± SEM, and were analyzed by one-way ANOVA followed by a Tukey post-hoc test for multiple comparisons using Prism 5 software. For all statistical comparisons, the variance among the groups was similar as determined by a Bartlett’s test for equal variance.

## Additional Information

**How to cite this article**: Jiang, J. *et al.* Hydrogen Sulfide–Mechanisms of Toxicity and Development of an Antidote. *Sci. Rep.* **6**, 20831; doi: 10.1038/srep20831 (2016).

## Supplementary Material

Supplementary Information

## Figures and Tables

**Table 1:** Intracellular ATP Concentration.

Time	hiPSC-Neurons			Human Foreskin Fibroblasts		
	Control	NaSH	KCN	Control	NaSH	KCN
(min)			(nmol/10^6^ cells)			
0	5.16 ± 0.9	—	—	5.92 ± 0.9	—	—
30	—	5.58 ± 1.6	5.03 ± 1.2	—	5.62 ± 1.1	5.88 ± 1.3
60	—	4.32 ± 1.3	5.24 ± 0.8	—	5.83 ± 0.7	5.31 ± 1.9
120	5.07 ± 1.4	4.39 ± 1.7	4.67 ± 1.0	5.67 ± 0.8	5.05 ± 1.2	5.74 ± 1.1

Human inducible pluripotent stem cell-derived neurons or primary human fibroblasts were incubated with 1 mM sodium hydrogen sulfide (NaSH) or 1 mM potassium cyanide (KCN) for the indicated times. They were then extracted rapidly in acid, and the intracellular ATP concentration was measured using firefly luciferase. The ATP concentration showed no significant change in either cell type under any condition. The data are mean ± SEM of three independent experiments performed in triplicate on separate days.
