# Supplementary material for: Hydrogen Sulfide—Mechanisms of Toxicity and Development of an Antidote
Source: Sci Rep. 2016 Feb 15;6:20831. doi: 10.1038/srep20831 (PMC4753484; doi:10.1038/srep20831)
Supplement: Supplementary Information [file srep20831-s1.pdf]

SUPPLEMENTARY INFORMATION

Hydrogen Sulfide—Mechanisms of Toxicity and Development of an Antidote

Jingjing Jiang[1], Adriano Chan[1], Sameh Ali[2-4], Arindam Saha[1], Kristofer J. Haushalter[5], Macrina Lam[1], Megan Glasheen[1], James Parker[6], Matthew Brenner[7], Sari B. Mahon[7], Hemal H. Patel[2,3], Rajesh Ambasudhan[6], Stuart A. Lipton[6,8], Renate B. Pilz[1], and Gerry R. Boss[1]

Departments of Medicine[1], Anesthesiology[2], Chemistry and Biochemistry[5], and Neurosciences[8], University of California San Diego, La Jolla, California, USA

[3]VA San Diego Healthcare System, San Diego, California, USA

[4]Center for Aging and Associated Diseases, Helmy Institute of Medical Sciences, Zewail City of Science and Technology, Giza, Egypt

[6]Neurodegenerative Disease Center, The Scintillon Institute, San Diego, California, USA

[7]Beckman Laser Institute, University of California Irvine, Irvine, California, USA

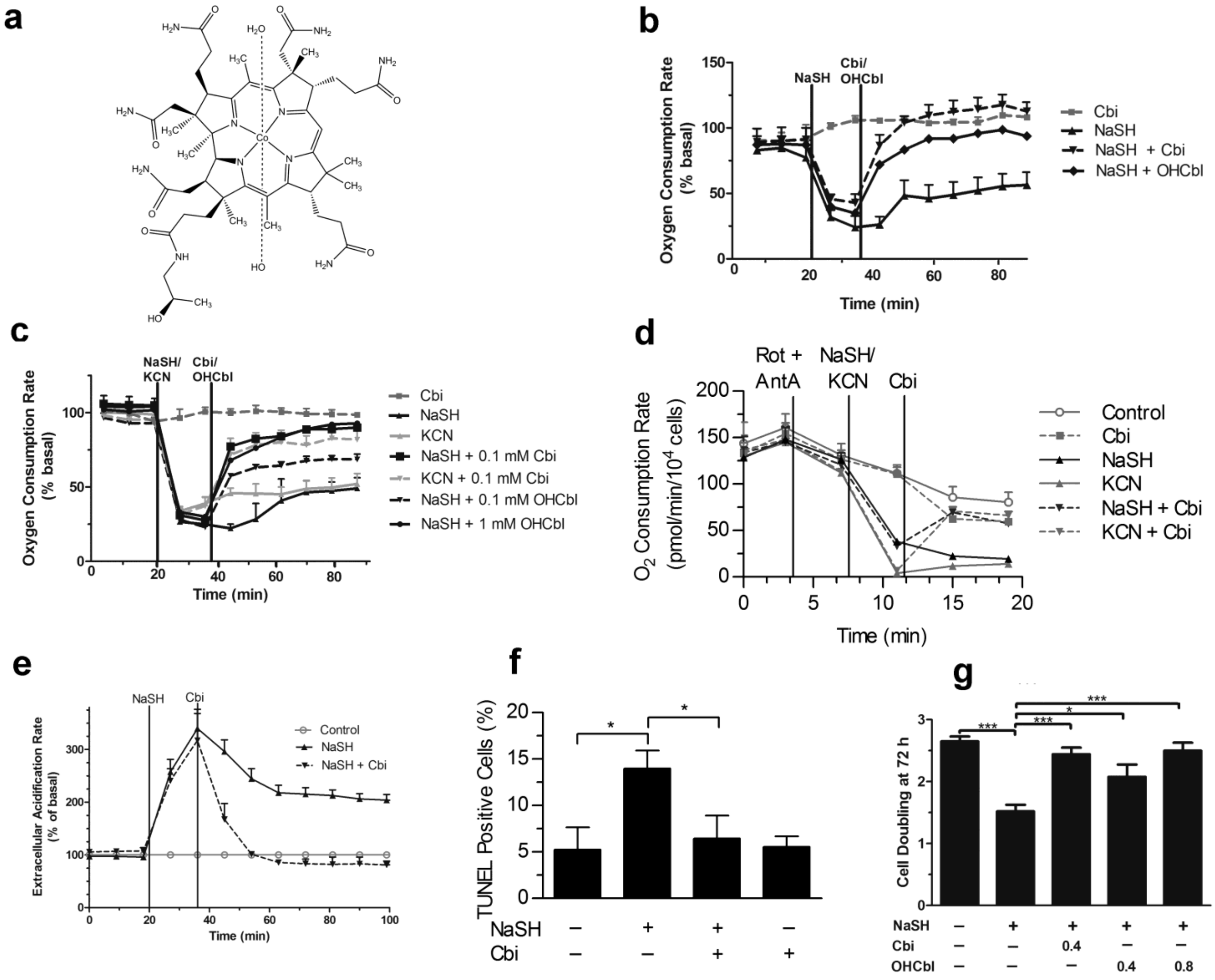

Supplemental Figure 1. (**a**). Aquohydroxocobinamide structure. (**b**,**c**). Oxygen consumption rate of primary human fibroblasts (**b**) or Cos-7 cells (**c**) is expressed as percent of control cells. At the times indicated by the vertical lines, 1 mM NaSH or 1 mM KCN and 0.1 mM cobinamide (Cbi) or 0.1/1 mM hydroxocobalamin (OHCbl) were added to the cells as indicated. NaSH-treated cells (triangles, solid black line); cobinamide-treated cells (squares, dashed grey line); NaSH-treated cells subsequently treated with cobinamide (inverted triangles, black line in **b**, and black squares in **c**); KCN-treated cells (triangles, solid grey line), KCN-treated cells subsequently treated with cobinamide (inverted triangles, dashed grey line); NaSH-treated cells subsequently treated with 0.1 mM hydroxocobalamin (diamonds in **b** and inverted triangles with dashed line in **c**); and NaSH-treated cells subsequently treated with 1 mM hydroxocobalamin (circles in **c**). (**d**). Oxygen consumption rate of permeabilized Cos-7 cells was measured in the presence of the complex IV substrate TMPD. At the indicated times, 2 µM rotenone and 4 µM antimycin A—complex I and III inhibitors, respectively, 0.5 mM NaSH or 0.5 mM KCN, and/or 100 µM cobinamide were added to the cells. Symbols same as in (**b**,**c**); non-treated control cells (open circles, solid grey line). (**e**). Simultaneous with the measurement of the oxygen consumption rate in Cos-7 cells (**c**), the extracellular acidification rate was measured. Non-treated cells (open circles); NaSH-treated cells (triangles); NaSH-treated cells subsequently treated with cobinamide (inverted triangles, dashed lines). (**f**,**g**). Primary rat cortical neurons (**f**) or Cos-7 cells (**g**) received 1 mM NaSH as indicated for 2 h, with 0.4 mM cobinamide or 0.4 or 0.8 mM hydroxocobalamin added to some cells during the second hour; the cells were washed and placed in fresh medium. (**f**). At 20 h, the cells were fixed and stained *in situ*; numbers indicate the mean ± SEM of TUNEL-positive cells in six areas subjected to densitometric scanning. (**g**). Number of cell doublings at 72 h is shown. Excluding Panel **a**, the data in all panels are the means ± SEM of three independent experiments performed in triplicate on separate days. In Panels **b**-**e**, the difference between the cobinamide-treated and the NaSH or KCN only condition was significant within 8 min post cobinamide addition ($P<0.05$). In Panels **f** and **g**, *$P<0.05$ and ***$P<0.001$ for indicated comparisons.

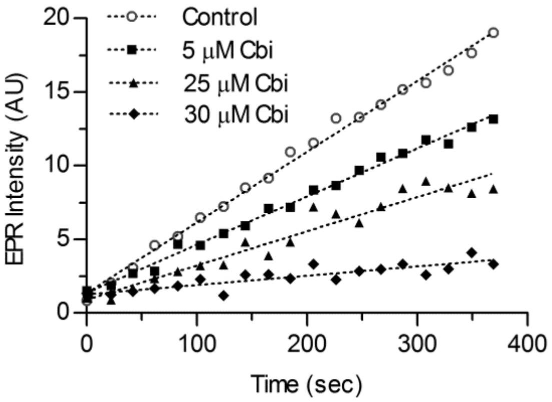

Supplemental Figure 2. Superoxide anion was generated using a hypoxanthine-xanthine oxidase system. Increasing amounts of cobinamide (Cbi) were added to the system, and DEPMPO was used as a spin trap to detect production of free superoxide anion via EPR. Control, circles; 5 µM Cbi, squares; 25 µM Cbi, triangles; 30 µM Cbi, diamonds. The data are the means of triplicate samples from a representative experiment; two additional independent experiments were performed with similar results.

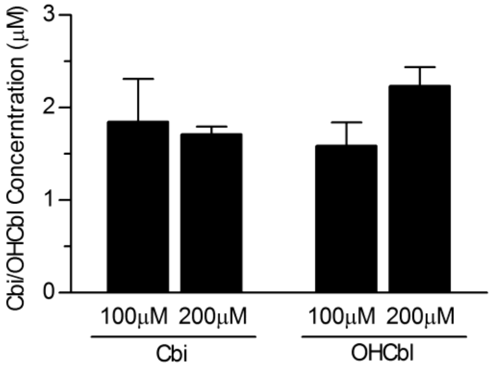

Supplemental Figure 3. Flies were grown on food containing the indicated concentrations of cobinamide (Cbi) or hydroxocobalamin (OHCbl). Sixty flies for each condition were decapitated, and the concentrations of cobinamide and hydroxocobalamin were quantified in the bodies by high performance liquid chromatography. The data are the means ± SEM of three independent experiments performed on separate days. No statistically significant differences were found among the four conditions.
